# Who Prepares More for End of Life? The Factors Associated With End-of-Life Planning Among Older Adults

**DOI:** 10.1093/geroni/igaa057.1360

**Published:** 2020-12-16

**Authors:** Hayoung Park, Hey Jung Jun, Susanna Joo, Sun Ah Lee

**Affiliations:** Yonsei University, Seoul, Republic of Korea

## Abstract

Being an “aged” society, only recently in Korea have there been growing interests in well-dying and end-of-life planning. Therefore, the purpose of this study was to investigate the factors associated with the level of end-of-life planning among Korean older adults. For the analyses, 2017 National Survey of Older Koreans was utilized and the sample was 10,073 older adults aged 65 and above. The independent variables were age, gender, employment, education level, marital status, household income, religion, number of children, and life satisfaction. The dependent variable, the level of end-of-life planning, was measured as the sum of five items of the question asking whether they have prepared the following items in preparation for end-of-life: shroud (8.3% prepared), cemetery/charnel house (25.1% prepared), mutual aid society (13.7% subscribed), will (0.5% prepared), lectures in preparation for end-of-life (0.4% participated). The results from multiple regressions using STATA 15.0 indicated that the level of end-of-life planning was significantly associated with age (b=.02, p<.001), gender (b=-.07, p<.001), education level (b=.02, p<.01), household income (b=2.56, p<.001), religion (b=.06, p<.001), number of children (b=.04, p<.001), and life satisfaction (b=.17, p<.001). That is, they have planned more for their end-of-life when older in age, female, more educated, wealthier, with religion, had more children, and more satisfied with life. The findings from this study suggest that political interventions are required to promote end-of-life planning education especially for less educated older adults with low income for their “well-dying”. Further researches are needed to explore other personal/interpersonal factors associated with end-of-life planning.

